# Role of Flexibility in Protein-DNA-Drug Recognition: The Case of Asp677Gly-Val703Ile Topoisomerase Mutant Hypersensitive to Camptothecin

**DOI:** 10.1155/2012/206083

**Published:** 2012-01-19

**Authors:** Ilda D'Annessa, Cinzia Tesauro, Paola Fiorani, Giovanni Chillemi, Silvia Castelli, Oscar Vassallo, Giovanni Capranico, Alessandro Desideri

**Affiliations:** ^1^National Research Council (CNR) and Department of Biology, University of Rome Tor Vergata, Via Della Ricerca Scientifica, Rome 00133, Italy; ^2^CASPUR, Via dei Tizii 6b, Rome 00185, Italy; ^3^Department of Biochemistry “Giovanni Moruzzi,” University of Bologna, Via Irnerio 48, 40126 Bologna, Italy

## Abstract

Topoisomerases I are ubiquitous enzymes that control DNA topology within the cell. They are the unique target of the antitumor drug camptothecin that selectively recognizes the DNA-topoisomerase covalent complex and reversibly stabilizes it. The biochemical and structural-dynamical properties of the Asp677Gly-Val703Ile double mutant with enhanced CPT sensitivity have been investigated. The mutant displays a lower religation rate of the DNA substrate when compared to the wild-type protein. Analyses of the structural dynamical properties by molecular dynamics simulation show that the mutant has reduced flexibility and an active site partially destructured at the level of the Lys532 residue. These results demonstrate long-range communication mechanism where reduction of the linker flexibility alters the active site geometry with the consequent lowering of the religation rate and increase in drug sensitivity.

## 1. Introduction

Human DNA topoisomerase I (hTop1) catalyzes the relaxation of supercoiled DNA through the transient cleavage of one strand of a DNA duplex and is fundamental to processes such as replication, recombination, and transcription [1–3]. The enzyme is composed of 765 amino acids, and the crystal structure of the N-terminal truncated protein (topo70) together with proteolytic experiments has shown that the enzyme is composed of four different domains: the NH_2_-terminal domain (residues 1–214), the core domain (215–635), the linker domain (636–712), and the COOH-terminal domain (713–765) [4–6]. Changes in DNA topology are achieved by introducing a transient break of the phosphodiester bond of one strand in the duplex DNA. The phosphodiester bond energy is preserved during catalysis through the formation of a transient covalent phosphotyrosine bond between the catalytic Tyr723 and the 3′-end of the broken DNA strand. After changing the linking number a second nucleophilic attack, driven by the 5′-hydroxyl DNA end, restores an intact double-stranded DNA, and the enzyme is released [6].

Eukaryotic topoisomerase I is the target of the antitumor drug camptothecin (CPT), which reversibly stabilizes the cleavable intermediate complex formed in the catalytic cycle of the enzyme, slowing the religation step of the enzyme. The stalled topoisomerase I may then collide with the progression of the replication fork producing lethal double-strand DNA breaks and cell death [7]. An important contribution toward the understanding of the interaction of CPT with topoisomerase I and DNA was provided by the crystal 3D structure of the ternary complex between topo70 covalently linked to DNA and the CPT derivative topotecan (TPT) [8]. The structure shows that the drug intercalates into the DNA duplex and moves the 5′-hydroxyl end of the DNA away from the scissile phosphate. This misalignment of the two ends likely slows down the religation step [8]. In addition to the effects on the religation reaction, CPT binding also reduces the linker domain mobility [9], as recently confirmed by molecular dynamics simulation of the ternary hTop1-DNA-TPT complex [10]. The presence of the drug affects not only the mobility of the linker domain but also the geometry of the active site, being the drug in direct interaction with Lys532 for all the simulation time [10]. This long-range effect results in linker domain showing a defined electron density in the structure of the topotecan-DNA-topo70 ternary complex, but not in that of the DNA-topo70 binary complex crystallized in the same conditions [8].

 In a previous report [11], the presence of two mutations (Asp677Gly and Val703Ile) in the linker domain was shown to confer increased CPT sensitivity to the hTop1 in yeast cells. The presence of the linker domain has been shown to be required for full inhibition of hTop1 by CPT [12]. In the case of the mutation Ala653Pro that confers a drug resistance phenotype to Top1, a combined experimental and simulative approach demonstrated that drug resistance is associated with increased linker mobility, which affects the efficiency of the religation process [9]. A perturbed linker dynamics coupled to a reduced religation rate has been also reported for the Lys681Ala-linker-located mutation [13] and a correlation between changes in linker flexibility and the enzyme specific activity has been proposed based on the characterization of the Ala653Pro-Thr718Ala double mutant [14]. Therefore, the current knowledge suggests that mutations in the linker domain can affect DNA binding and CPT sensitivity of hTop1, although the exact mechanism remains to be elucidated.

 In the present paper, we have investigated the Asp677Gly-Val703Ile double mutant to find a molecular explanation for its CPT hypersensitivity. Through a combined experimental and molecular dynamics approach, we provide evidence that the CPT-hypersensitive double mutant displays a lower religation rate due to a displacement in the active site of the Lys532 residue. Moreover, the mutant displays a lower linker flexibility confirming the crucial role of this domain in controlling the drug sensitivity of human Top1.

## 2. Experimental Procedures

### 2.1. Yeast Strain and Plasmids

Camptothecin was dissolved in Me_2_SO to a final concentration of 4 mg/mL and stored at 20°C. Anti-FLAGM2 affinity gel, FLAG peptide, and M2 monoclonal antibody were purchased from Sigma. *Saccharomyces cerevisiae* strain *EKY3* (*ura3–52, his3∆200, leu2∆1, trp1∆63, top1::TRP1, MAT*α**) was described previously [15, 16]. Plasmid YEpGAL1-wild-type in which the human topoisomerase I is expressed under the galactose inducible promoter in a multicopy plasmid was described previously [17]. Asp677Gly-Val703Ile was generated by oligonucleotide-directed mutagenesis of the YEpGAL1-wild-type. The epitope-tagged construct YEpGAL1-e-wild-type contains the N-terminal sequence FLAG: DYKDDDDY (indicated with “e”), recognized by the M2 monoclonal antibody. The epitope tag was subcloned into YEpGAL1-Asp677Gly-Val703Ile to produce YEpGAL1-e-Asp677Gly-Val703Ile.

### 2.2. Purification of DNA Topoisomerase I

To purify the topoisomerase I epitope-tagged EKY3 cells were transformed with YEpGAL1-e-wild-type and YEpGAL1-e-Asp677Gly-Val703Ile, grown on SC-uracil plus 2% dextrose and diluted 1:100 in SC-uracil plus 2% raffinose. At an optical density A_595_ of 1.0, the cells were induced with 2% galactose for 6 h. Cells were then harvested by centrifugation, washed with cold water, and resuspended in 2 mL buffer/g cells using a buffer containing 50 mM Tris, pH 7.4, 1 mM EDTA, 1 mM ethylene glycol-bis(2-aminoethylether)-N, N, N′, N′-tetraacetic acid, 10% (v/v) glycerol completed with protease inhibitors cocktail (Roche 1836153) and supplemented with 0.1 mg/mL sodium bisulfite and 0.8 mg/mL sodium fluoride. After addition of 0.5 volume of 425–600 *μ*m diameter glass beads the cells were disrupted by vortexing for 30 s alternating with 30 s on ice. The lysate was centrifuged and KCl (final concentration 0.15 M) was added to the sample prior to loading onto 2 mL ANTI-FLAG M2 affinity gel column equilibrated as described in the technical bulletin (Sigma). The column was washed with 20 column volumes of TBS (50 mM Tris HCl, 150 mM KCl, pH 7.4). Elution of FLAG-fusion topoisomerase I was performed by competition with five column volumes of a solution containing 100 *μ*g/mL FLAG peptide in TBS. Fractions of 500 *μ*L were collected and glycerol (final concentration 40%) was added; all preparations were stored at –20°C. The fractions were resolved by SDS-polyacrylamide gel electrophoresis; protein concentration and integrity were measured through immunoblot assay, using the epitope-specific monoclonal antibody M2. After normalization to protein content, the activity of the wild-type and mutant DNA topoisomerase I, as assayed by relaxation of supercoiled DNA in 150 mM KCl, was found to be almost identical. In all the biochemical experiments the same amount of wild-type and mutated protein has been used.

### 2.3. Cleavage/Religation Equilibrium

The 25 mer oligonucleotide, CL25 (5′-GAAAAAAGACTTAGAAAAATTTTTA-3′), was radiolabeled with [*γ*-^32^P]ATP at its 5′ end. The CP25 complementary strand (5′-TAAAAATTTTTCTAAGTCTTTTTTC-3′) was phosphorylated at its 5′ end with unlabeled ATP. The two strands were annealed at a 2-fold molar excess of CP25 over CL25. A final concentration of 20 nM duplex CL25/CP25 was incubated with an excess of enzyme at 25°C in 20 mM Tris pH 7.5, 0.1 mM Na_2_EDTA, 10 mM MgCl_2_, 50 *μ*g/mL acetylated BSA, and 150 mM KCl, in the presence or absence of different concentrations of CPT. After 30 minutes, the reaction was stopped by adding 0.5% SDS and the sample was digested with trypsin after precipitation with ethanol. Reaction products were resolved in 16% acrylamide—7 M urea gels, and the percentage of cleavage (% Cl) was determined by PhosphorImager and ImageQuant software.

### 2.4. Kinetics of Cleavage Using Oligonucleotide Substrate

Oligonucleotide substrate CL14 (5′-GAAAAAAGACTTAG-3′) was radiolabeled with [*γ*-^32^P]ATP at its 5′ end. The CP25 complementary strand (5′-TAAAAATTTTTCTAAGTCTTTTTTC-3′) was phosphorylated at its 5′ end with unlabeled ATP. The two strands were annealed at a 2-fold molar excess of CP25 over CL14 as previously described [18]. The suicide cleavage reactions were carried out by incubating 20 nM of the duplex with an excess of enzyme in 20 mM Tris pH 7.5, 0.1 mM Na_2_EDTA, 10 mM MgCl_2_, 50 *μ*g/mL acetylated BSA, and 150 mM KCl at 23°C in a final volume of 50 *μ*L as described by Yang and Champoux [18]. A 5 *μ*L sample of the reaction mixture was removed before addition of the protein and used as the zero time point. At various time points 5 *μ*L aliquots were removed and the reaction stopped with 0.5% SDS. After precipitation with ethanol samples were resuspended in 5 *μ*L of 1 mg/mL trypsin and incubated at 37°C for 30 min. Samples were analyzed by denaturing urea/polyacrylamide gel electrophoresis. The percentage bound cleavage product was determined by PhosphorImager and ImageQuant software and normalized on the total amount of radioactivity in each lane.

### 2.5. Kinetics of Religation Using Oligonucleotide Substrate

20 nM CL14/CP25, prepared as described above, was incubated with an excess of enzyme for 60 min at 23°C followed by 30 min at 37°C in 20 mM Tris pH 7.5, 0.1 mM Na_2_EDTA, 10 mM MgCl_2_, 50 *μ*g/mL acetylated BSA, and 150 mM KCl. Religation reactions were initiated by adding a 200-fold molar excess of R11 oligonucleotide (5′-AGAAAAATTTT-3′) over the duplex CL14/CP25. At various time points 5 *μ*L aliquots were removed and the reaction stopped with 0.5% SDS. After ethanol precipitation samples were resuspended in 5 *μ*L of 1 mg/mL trypsin and incubated at 37°C for 30 min. Samples were analyzed by denaturing urea/polyacrylamide gel electrophoresis. The percentage of the remaining covalent complex was determined by PhosphorImager and ImageQuant software and normalized on the total amount of radioactivity in each lane.

### 2.6. MD Simulation

The DNA-Topo70 covalent complex, without the N-terminal domain, has been modelled obtaining the starting positions from the X-ray structure of 1a36 for residues 215–633 and 641–765 [6], and from the X-ray structure 1ej9 for residues 203–214 [19]. The five residues 636–640 that form the loop connecting the linker domain and the C-terminal domain, lacking in the 1a36 structure in the Protein Data Bank (PDB http://www.rscb.org/pdb/), and the covalent bond between the catalytic Tyr723 and the −1 base, Thy10, have been added to the structure via molecular modelling. The double mutant, obtained substituting Asp677 and Val703 with a glycine and an isoleucine, respectively, has been minimized with the Powell method implemented in the SYBYL program (Tripos, St. Louis, MO), to establish the correct three-dimensional positioning of the added residues and to normalize the stoichiometry of the structures. The two systems were modelled with the leaprc.ff03 force field of AMBER version 8.0 [20] and solvated with TIP3P water molecules. Na^+^ counterions have been added to neutralize the systems. The resulting systems were composed of 9417 protein atoms, 1401 DNA atoms, 21 Na^+^ atoms, and 35193 water molecules for the native protein, for a total amount of 116418 atoms; 9415 protein atoms, 1401 DNA atoms, 20 Na^+^ atoms, and 39824 water molecules for the mutant, for a total amount of 121056 atoms.

The two systems have been simulated for 10000 ps each, using AMBER version 8.0 with periodic boundary conditions, applying a *cut-off* of 9 Å for nonbonding interactions and updating neighbouring list every 10 steps. The electrostatic interactions were taken into account by means of the particle mesh Ewald method [21, 22], and the SHAKE algorithm [23] was used to restrict the length of hydrogen bonds. Solvent and ions were first optimized and relaxed keeping the solute atoms constrained to their starting positions, using a decreasing force of 500, 25, 15, and 5 kcal/(mol Å). Systems were simulated at a constant temperature of 300 K using the method of Ryckaert et al. [24] and at a constant pressure of 1 bar with a 2.0 fs time step. Temperature and pressure coupling constants were fixed at 0.5 ps.

The analyses have been carried out on the last 10000 ps, eliminating the equilibration step, which correspond to the first nanosecond. Hydrogen bonds, standard deviations (RMSD), and fluctuations (RMSF) have been calculated with the GROMACS MD package version 3.2.1 [25] (http://www.gromacs.org/). The cut-off radius for direct hydrogen bond distance was fixed at 3.5 Å, and the angle was fixed at 120°. The graphs were obtained with the Xmgrace program (http://plasma-gate.weizmann.ac.il/Grace/) and the images with VMD version 1.8.5 [26] (http://www.ks.uiuc.edu/Research/vmd/).

## 3. Results

### 3.1. Effect of CPT on the Cleavage-Religation Equilibrium

**Figure 1:**
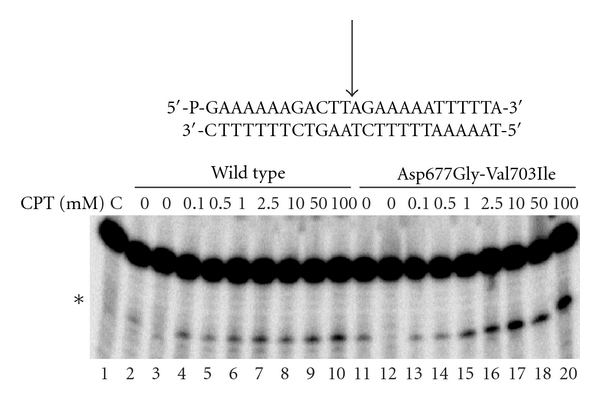
Gel electrophoresis of the products coming from the incubation of the wild-type topoisomerase I with the [*γ*-^32^P] end-labelled duplex DNA, shown at the top of the figure in the absence (lanes 2-3) or presence (lanes 4–10) of increasing amounts of CPT. The arrow at the DNA sequence indicates the CL1 site preferred by the wild-type protein. Lanes 11 and 12 and 13–19 show the same experiment with the Asp677Gly-Val703Ile mutant.

To investigate the level of CPT hypersensitivity of the Asp677Gly-Val703Ile mutant, the stability of the covalent DNA-enzyme complex was studied using the 25mer full duplex oligonucleotides substrate CL25 (5′-GAAAAAGACTTAGAGAAAAATTTT-3′)/CP25 5′-TAAAAATTTTTCTAAGTCTTTTTTC-3′). After 30 minutes of incubation in the absence or presence of increasing concentrations of CPT, the reaction products were analyzed by polyacrylamide-urea gel electrophoresis (Figure 1). The results confirm that the double mutant is more sensitive to CPT since the cleavage-religation equilibrium constant (K_eq_) is shifted toward cleavage than the wild-type protein (cf. lanes 7–10 to 16–19). A slight shift toward cleavage can also be inferred for the mutant in the absence of CPT, being the band due to the cleavable complex slightly detectable (lane 11). Thus, the DNA oligomer cleavage data support a change of the enzyme sensitivity to CPT due to the two linker mutations, in agreement with previous findings [11].

### 3.2. Cleavage and Religation Rate of Top1 and Top1 Double Mutant

**Figure 2:**
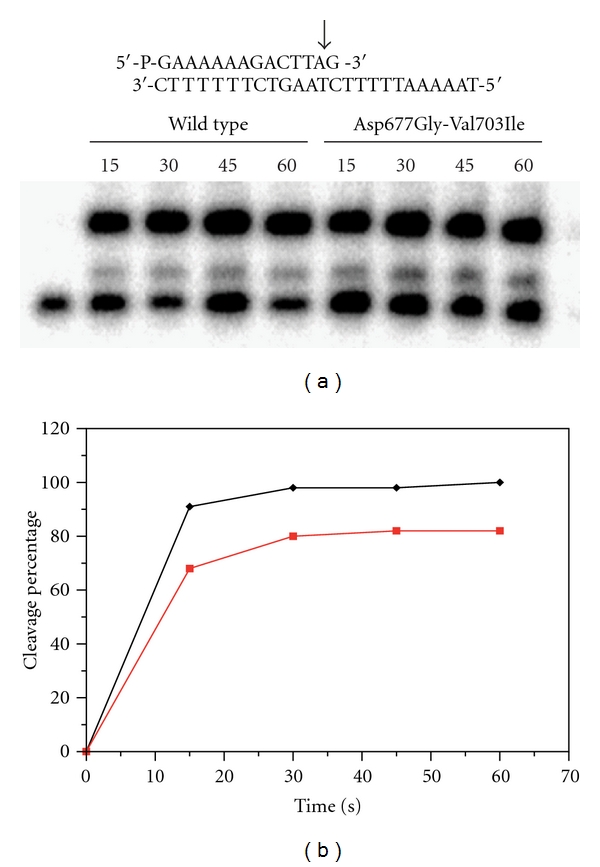
(a) Time course of the suicide cleavage reaction carried out with the substrate described at the top of the figure. The arrow indicates the preferential cleavage site for the wild-type protein. (b) Percentage of the cleaved DNA substrate plotted as a function of time for the wild-type (black line) and Asp677Gly-Val703Ile (red line) mutant.

 The DNA cleavage equilibrium constant K_eq_ is defined by k_cl_/k_rl_, that is, the ratio between cleavage and religation rates. We have analyzed the single rates of the enzyme catalytic cycle to shed light on the different behaviours of wild-type and mutant proteins. To investigate the effect of the double mutation on the cleavage reaction, a 5′ end radiolabeled suicide substrate CL14 (5′-GAAAAAGACTTAG-3′) has been annealed to the CP25 (5′-TAAAAATTTTTCTAAGTCTTTTTT-3′) complementary strand to produce a duplex with an 11-base 5′ single-strand extension (Figure 2(a)). With this substrate the religation step is abolished because the short oligonucleotide generated during cleavage cannot be religated, leaving the enzyme covalently attached to the 3′ end [18]. The suicide cleavage substrate has been incubated with an excess of native and mutated protein in a time course experiment. The amount of cleaved fragment, normalized to the plateau value of the topoisomerase, is plotted as a function of time in Figure 2(b). The data show that both enzymes efficiently cleave the suicide substrate at physiological ionic strength, reaching a plateau level in approximately the same time.

**Figure 3:**
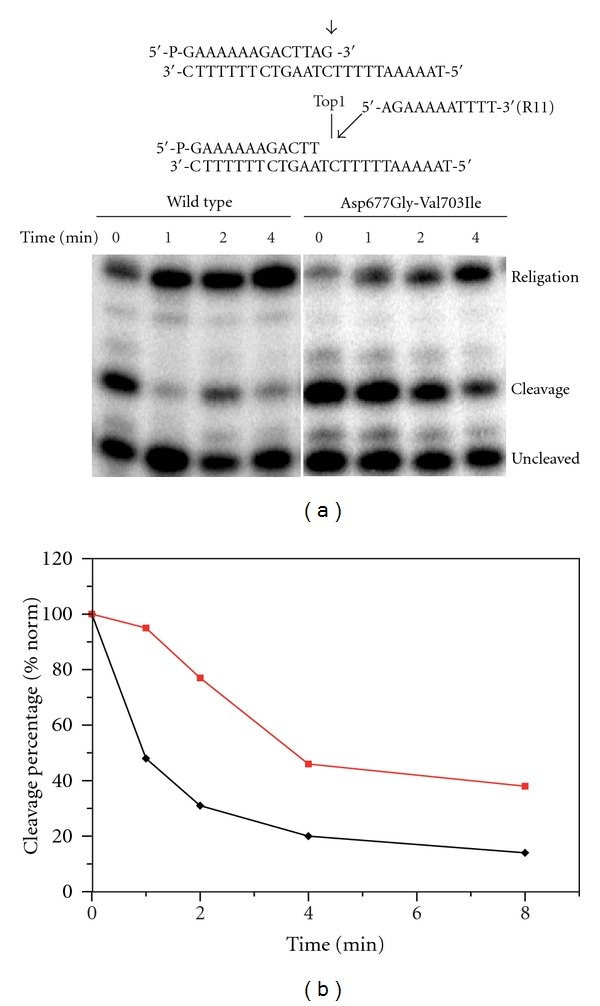
(a) Time course of the religation experiment between the R11 substrate and the covalent complex of the wild-type or Asp677Gly-Val703Ile mutant. (b) Percentage of the remaining covalent complex, plotted at different times for the wild-type enzyme (black line) and the Asp677Gly-Val703Ile mutant (red line).

The chemical step of DNA ligation was studied assaying the ability of both enzymes to religate the oligonucleotide R11 (5′-AGAAAAATTTT-3′) once added to the precleaved suicide substrate. The first step of the reaction consists in the incubation of an excess of native and mutated enzyme with the suicide substrate for sixty minutes in order to generate the cleaved complex having the enzyme covalently attached to the 3′ end. Once cleavage has occurred, the R11 oligonucleotide is added to the mixture to initiate the ligation process. Aliquots were removed at different times, the reaction stopped by addition of SDS, and the products analyzed by polyacrylamide gel electrophoresis (Figure 3(a)). The percentage of the remaining cleavable complex, determined as described in Section 2, has been plotted as a function of time in Figure 3(b). The results show that the religation rate is much slower for the mutant than for the wild-type, indicating that mutation of the 677 and 703 residues of the linker domain decreases the religation process likely whereas the cleavage process is practically unaffected.

### 3.3. Molecular Dynamics Simulation

 The observation that the main effect of the double mutation is the reduction of the religation rate suggests modified rearrangement of the active site. In order to verify this hypothesis we have carried out a 10 ns molecular dynamical simulation of the mutated system and compared it with an identical simulation carried out for the native enzyme.

**Figure 4:**
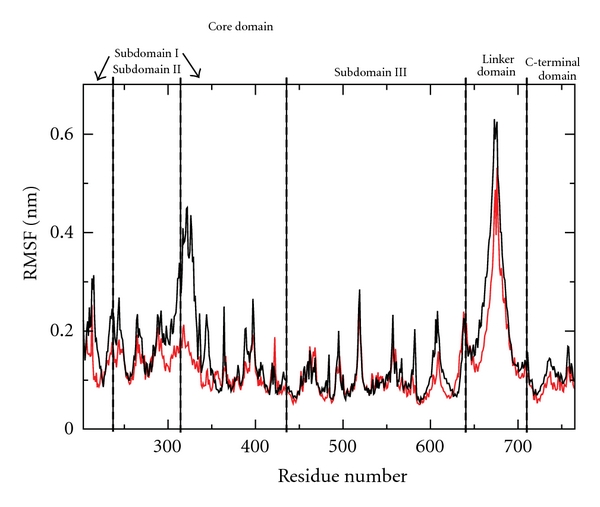
Averaged per-residue RMSF represented as a function of the hTop1 residue number for the native (black line) and mutated (red line) enzyme.

**Figure 5:**
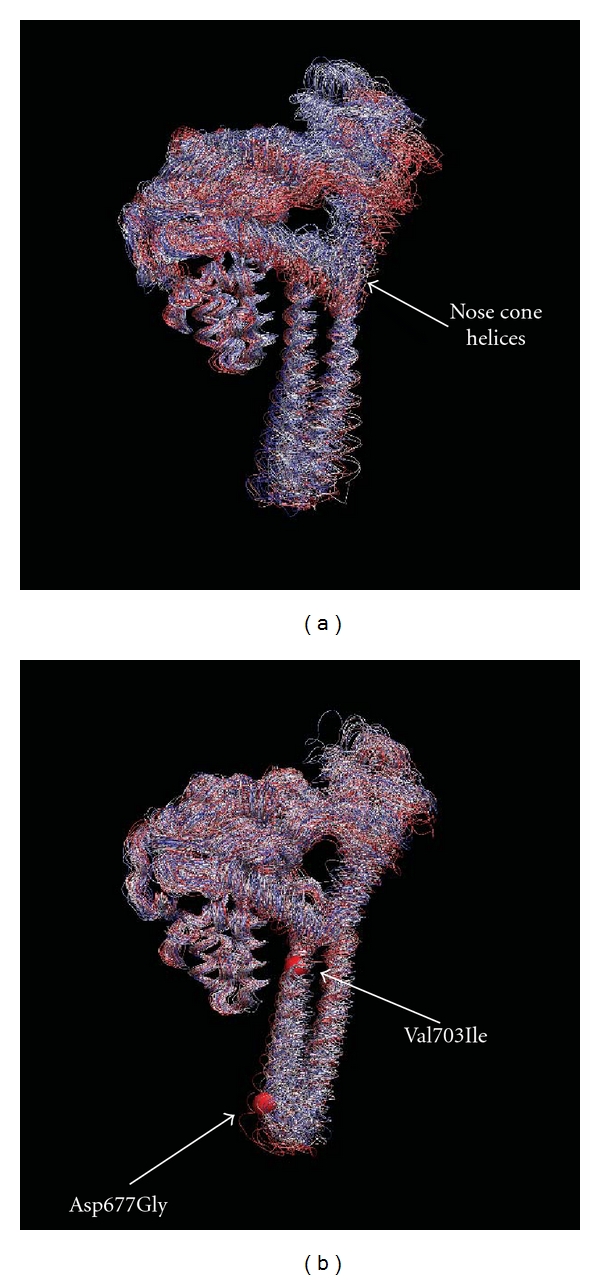
Overlap of 50 snapshots extracted every 200 ps for the wild-type (a) and the double mutant enzyme (b) trajectories. The structures have been superimposed on the C*α* atoms of the active site residues Arg488, Arg590, and Tyr723. The “nose cone helices” are indicated by an arrow. The sites of the two mutations on the linker domain are highlighted by a red sphere in the mutant structure.

 The RMSD value for residues of the native and mutated proteins as a function of time, calculated after a mass weighted superposition on the starting structure, indicates that for both systems the deviation from the starting structure is mainly due to oscillation of the linker domain since upon its elimination the RMSDs are stabilized (data not shown). The deviation is smaller for the mutant (about 0.2 nm) than for the wild-type (about 0.3 nm) indicating that the mutations reduce the conformational space sampled by the protein (data not shown). Analysis of the per-residue root mean square fluctuation, RMSF, indicates a similar behaviour, with the linker domain being the most fluctuating region in both proteins (Figure 4). However, the double mutant shows a lower degree of fluctuation for each domain when compared to the wild-type protein, and this is more evident for the linker domain (residues 636–712) and for the core domain, at the level of the “nose cone helices” (residues 300–340). The different degree of flexibility can be appreciated by overlapping the snapshots extracted from the two dynamics taken every 200 ps as shown in Figure 5. The wild-type protein (Figure 5(a)) samples a conformational space larger than that observed for the double mutant (Figure 5(b)). This is particularly evident for the linker domain and the “nose cone helices,” where the different degrees of fluctuation make the cavity that accommodates the DNA larger in the mutant than that observed in the wild-type.

**Figure 6:**
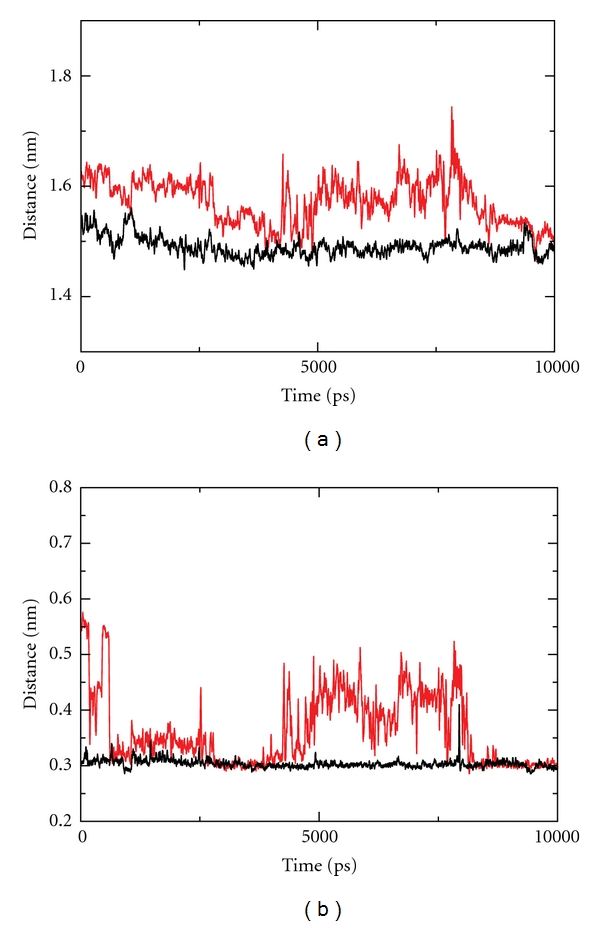
Atomic distance as a function of time between Lys532N*ξ* : Tyr723O*δ* (a) and Lys532N*ξ* : Ade11O5′ (b). Both distances have been mediated every 50 frames. The black line and red traces represent the native protein and mutant protein data, respectively.

 The region around the active site has been analyzed in detail to detect possible differences that might be correlated with the experimentally observed reduction of the mutant religation rate. Analysis of the time dependence of the relative distances of the residues forming the catalytic pentad indicates a structural-dynamical reorientation in the double mutant, mainly at the level of Lys532. In the wild-type protein the amino group of the lateral chain of Lys532 maintains a constant distance from the catalytic Tyr723 and from the +1 adenine base (Ade11) of the scissile strand over the entire simulation time, as shown in Figures 6(a) and 6(b), black traces, in the latter case an almost constant value of 0.3 nm is found. For the mutant these distances are longer and much more fluctuating (Figures 6(a) and 6(b), red traces). In detail a high degree of fluctuation is observed for the Lys532-Ade11 distance, ranging from 0.3 to 0.6 nm. In the case of the vaccinia topoisomerase it has been proposed that the lateral chain of Lys167 (corresponding to human Lys532) would accept a proton from the attacking 5′-OH group of the +1 base in the religation step [27, 28]. A long and fluctuating 5′OH-N*ζ*Lys532 distance, as that observed in Figure 6(b), renders the donation of the proton more difficult, providing an explanation for the lower religation rate of the mutant (Figure 3).

## 4. Discussion

The experimental cleavage-religation data reported in Figure 1 confirms that the Asp677Gly-Val703Ile double mutant is more sensitive to CPT than the wild type. The increase in CPT sensitivity is coupled to a reduction of the religation rate as shown in Figure 3. This result emphasizes the critical role of the linker domain and shows how mutations in this domain can have an effect on the catalytic site, that is, in a region far from the mutations, demonstrating the occurrence of communication between domains localized far away one from the other. The two mutations are in fact widely separated from the drug binding site, the first one, Asp677Gly, being located in the loop connecting the two linker *α*-helices, and the second one, Val703Ile, at the bottom of the second linker helix (Figure 6(b)). Molecular dynamics simulations provide some insight at the atomic level of the camptothecin hypersensitivity and of the reduced religation rate of the Asp677Gly-Val703Ile mutant. Analysis of the trajectories evidences a lower flexibility of the mutant when compared to the wild-type, in particular for the linker domain and the “nose cone helices” (Figures 4 and 5). In a previous study, aimed at the characterization of the CPT resistant single mutation Ala653Pro, the linker was found to display a flexibility larger than in the wild-type [9] whilst a reduced religation rate coupled to an altered flexibility has been found in the Lys681Ala mutant [13]. The different flexibility of the linker domain seems then to be directly correlated to the enzyme sensitivity to camptothecin and to the rate of the religation reaction. These findings are in line with the fact that the linker domain is observed in the X-ray diffraction of the crystal containing the ternary CPT-DNA-topoisomerase complex, but not in the binary DNA-topoisomerase complex [8], and a reduced linker mobility has been shown by an MD study of the hTop1-DNA-TPT ternary complex [10]. Likely, a high fluctuating linker domain induces a series of conformations that are less prone to interact with the CPT molecule.

Another interesting result of the analysis of the trajectories concerns the orientation and relative distance of the residues belonging to the catalytic pentad. Four of the five catalytic residues (Arg488, Arg590, His632, and Tyr723) display an almost identical behaviour in both the native and mutated enzyme. On the other hand, Lys532 in the mutant is more fluctuating and displays a large distance from both the +1 adenine base 5′ oxygen and the oxygen of Tyr723 (Figure 6). On the basis of studies carried out on the vaccinia enzyme the lysine residue has been proposed to accept a proton from the +1 base, downstream to the cleavage site of the scissile strand, and to transfer it, directly or through an arginine of the catalytic site, to the catalytic tyrosine, so promoting the religation of the scissile strand [27, 28]. The lengthening of this proton transfer (Figure 6(b)) may explain at atomic level the lowered religation rate observed in the mutant (Figure 3).

In conclusion, our study indicates that protein dynamics plays an important role in the protein-DNA-drug complex recognition. In detail the double Asp677Gly-Val703Ile mutation makes the enzyme hypersensitive to camptothecin inducing a reduction of the linker flexibility coupled to a slower religation rate, due to a partial destructuration of the active site and in particular of the lengthening of the distance between Lys532 and the +1 adenine base. Our study confirms the capability of the different domains to interact through long-range communication since mutations in the linker domain can affect regions of the protein far away, such as the active site region. These findings support a model where changes in linker flexibility alter the geometry of the active site with the consequent perturbation of the DNA cleavage/religation reaction catalyzed by hTop1.

## Abbreviations

Top1: DNA topoisomerase I
hTop1: Human DNA topoisomerase I
CPT: Camptothecin
BSA: Bovine serum albumin
SC: Synthetic complete medium
MD: Molecular dynamics.
